# Partial Characterization and Immunomodulatory Effects of Exopolysaccharides from *Streptococcus thermophilus* SBC8781 during Soy Milk and Cow Milk Fermentation

**DOI:** 10.3390/foods12122374

**Published:** 2023-06-15

**Authors:** Hajime Nakata, Yoshiya Imamura, Sudeb Saha, René Emanuel Lobo, Shugo Kitahara, Shota Araki, Mikado Tomokiyo, Fu Namai, Masanori Hiramitsu, Takashi Inoue, Keita Nishiyama, Julio Villena, Haruki Kitazawa

**Affiliations:** 1 Food and Feed Immunology Group, Laboratory of Animal Food Function, Graduate School of Agricultural Science, Tohoku University, Sendai 980-8572, Japan; hajime.nakata@pokkasapporo-fb.co.jp (H.N.); yoshiya.imamura.p8@dc.tohoku.ac.jp (Y.I.); sudeb.ds@sau.ac.bd (S.S.); k.s.coner.1525@gmail.com (S.K.); shota.araki.t4@dc.tohoku.ac.jp (S.A.); mikado.tomokiyo.t4@dc.tohoku.ac.jp (M.T.); fu.namai.a3@tohoku.ac.jp (F.N.); keita.nishiyama.a6@tohoku.ac.jp (K.N.); 2 Pokka Sapporo Food and Beverage Ltd., Nagoya 460-0008, Japan; masanori.hiramitsu@pokkasapporo-fb.co.jp (M.H.); takashi.inoue@pokkasapporo-fb.co.jp (T.I.); 3 Livestock Immunology Unit, International Education and Research Centre for Food and Agricultural Immunology (CFAI), Graduate School of Agricultural Science, Tohoku University, Sendai 980-8572, Japan; 4 Department of Dairy Science, Faculty of Veterinary, Animal and Biomedical Sciences, Sylhet Agricultural University, Sylhet 3100, Bangladesh; 5 Institute of Analytical Chemistry (Cátedra de Química Analítica III), Faculty of Biochemistry, Chemistry, and Pharmacy, National University of Tucumán, Tucuman 4000, Argentina; rene.lobo@fbqf.unt.edu.ar; 6 Laboratory of Immunobiotechnology, Reference Centre for Lactobacilli (CERELA-CONICET), Tucuman 4000, Argentina

**Keywords:** *Streptococcus thermophilus* SBC8781, exopolysaccharides, soy milk, cow milk, immunomodulatory activities, intestinal epithelial cells

## Abstract

The immunomodulatory properties of exopolysaccharides (EPSs) produced by *Streptococcus thermophilus* have not been explored in depth. In addition, there are no comparative studies of the functional properties of EPSs produced by streptococci in different food matrices. In this work, EPSs from *S. thermophilus* SBC8781 were isolated after soy milk (EPS-s) or cow milk (EPS-m) fermentation, identified, and characterized in their abilities to modulate immunity in porcine intestinal epithelial cells. Fresh soy milk and cow milk were inoculated with *S. thermophilus* SBC8781 (7 log CFU/mL) and incubated at 37 °C for 24 h. The extraction of EPSs was performed by the ethanol precipitation method. Analytical techniques, including NMR, UV-vis spectroscopy, and chromatography, identified and characterized both biopolymer samples as polysaccharides with high purity levels and similar Mw. EPS-s and EPS-m had heteropolysaccharide structures formed by galactose, glucose, rhamnose, ribose, and mannose, although with different monomer proportions. On the other hand, EPS-s had higher quantities of acidic polymer than EPS-m. The biopolymer production of the SBC8781 strain from the vegetable culture broth was 200–240 mg/L, which was higher than that produced in milk, which reached concentrations of 50–70 mg/L. For immunomodulatory assays, intestinal epithelial cells were stimulated with 100 µg/mL of EPS-s or EPS-m for 48 h and then stimulated with the Toll-like receptor 3 agonist poly(I:C). EPS-s significantly reduced the expression of IL-6, IFN-β, IL-8, and MCP-1 and increased the negative regulator A20 in intestinal epithelial cells. Similarly, EPS-m induced a significant reduction of IL-6 and IL-8 expressions, but its effect was less remarkable than that caused by EPS-s. Results indicate that the structure and the immunomodulatory activity of EPSs produced by the SBC8781 strain vary according to the fermentation substrate. Soy milk fermented with *S. thermophilus* SBC8781 could be a new immunomodulatory functional food, which should be further evaluated in preclinical trials.

## 1. Introduction

Exopolysaccharides (EPSs) secreted by food-grade microorganisms are considered “Generally Recognized as Safe” by the U.S.A. Food and Drug Administration and have been granted a “Qualified Presumption of Safety” status by the European Food Safety Authority. Therefore, EPSs produced by food-grade microorganisms are widely used to improve the rheology, texture, and sensory characteristics of various fermented products, such as yogurt and cheese [1,2,3]. Moreover, it was demonstrated that EPSs have several beneficial effects for health, including immunomodulatory [4,5,6], antitumor [7], antioxidant [8], antibacterial [9], and antiviral activities [10,11], as well as the capacity to diminish blood cholesterol levels [12]. Thus, the dairy/food industry is interested in the search for new EPSs that produce strains for the development of novel functional fermented products with beneficial properties for health. Among the food-grade microorganisms with the ability to produce EPSs, there is *Streptococcus thermophilus*, which is a Gram-positive lactic acid bacterium commercially used as starter culture for the manufacture of yogurt and cheese [5,11,13]. Of note, not all *S. thermophilus* strains can produce EPSs, and their beneficial properties were not studied in depth. In this regard, *S. thermophilus* SBC8781 has a remarkable capacity to produce EPS; however, there are no studies regarding the immunomodulatory abilities of this biopolymer.

EPS produced by *S*. *thermophilus* strains can modulate the systemic and mucosal immune responses. It was described that the purified EPS produced by *S. thermophilus* MN-BM-A01 protected the barrier integrity and differentially modulated the expression of pro-inflammatory factors in the intestinal mucosa after stimulation with dextran sulfate sodium or lipopolysaccharide (LPS) [14]. Similarly, EPSs producing the strains *S. thermophilus* ST1342, ST1275, and ST285 improved the strength of the barrier and restricted the adhesion and invasion of intestinal pathogens in the context of acute ulcerative colitis [15,16]. It was also shown that the EPS from *S. thermophilus* CRL1190 can decrease IFN-γ and increase IL-10 in the gastric mucosa of mice treated with acetyl-salicylic acid, an effect that was associated with the prevention of chronic gastritis development [17]. Furthermore, *S. thermophilus* CRL1190 EPS was shown to reduce the adhesion of *Helicobacter pylori* to gastric mucosa and decrease the expression of proinflammatory cytokines [18]. These findings indicate that EPSs derived from *S*. *thermophilus* can modulate immunity conferring protection against bacterial infections and detrimental inflammation. However, their impact on antiviral immunity was not explored.

Numerous fermented soy-based food products are consumed in Asian countries, including Korea, Japan, China, Indonesia, and Thailand [19]. Alongside dairy products, soy-based food products have become popular due to their various potential health benefits to the host because of their anticholesterolemic, hypolipidemic, and antiatherogenic properties [20]. Soy milk can serve as a low-cost non-dairy alternative with improved functional and nutritional properties. For example, soy milk can overcome the lactose intolerance problems in humans related to cow milk consumption [21]. However, the unpleasant bean flavor, together with the indigestible oligosaccharides, raffinose, and stachyose of soy milk, can be prone to the consumer acceptance. The fermentation of soy milk by lactic acid bacteria (LAB) has been suggested as an alternative to solve these problems [22,23]. Both soy milk and cow milk are considered excellent sources of nutrients for the growth of probiotic LAB due to the presence of amino acids, peptides, and oligosaccharides [24,25,26,27]. In addition, it was shown that LAB can produce EPS molecules during the fermentation of soy milk [28,29].

Considering the above facts, the aim of this work was to evaluate the ability of *S*. *thermophilus* SBC8781 to produce EPSs in both soy milk and cow milk. Furthermore, EPSs from *S. thermophilus* SBC8781 after soy milk or cow milk fermentation were identified and characterized in terms of their abilities to modulate an antiviral immune response in porcine intestinal epithelial (PIE) cells after the activation of the Toll-like receptor 3 (TLR3) signaling pathway. To the best of our knowledge, this is the first report to partially characterize the polymer structure and compare the EPS production by an *S. thermophilus* strain in soy milk and cow milk.

## 2. Materials and Methods

### 2.1. Bacteria, Culture Conditions, and Milk Fermentations

The *S. thermophilus* SBC8781 strain was provided by POKKA SAPPORO Food and Beverage Ltd. (Tokyo, Japan). The strain was stored at −80 °C in 20% glycerol solution until use. *S. thermophilus* SBC8781 was inoculated (1% *v*/*v*) in the whey of soy or cow milk supplemented with 0.5% yeast extract at 37 °C for 24 h.

Fresh soy milk was purchased by Fuji Oil Co., Ltd. (Osaka, Japan), and protein was removed by the addition of hydrochloric acid to the samples. *S. thermophilus* SBC8781 was inoculated (1% *v*/*v*) at a final cell density (7.0 log CFU/mL) in soy whey supplemented with 0.5% yeast extract. Commercially available cow milk from Meiji Co., Ltd. (Tokyo, Japan) was used and treated in the same manner. Soy milk and cow milk inoculated with the SBC8781 strain were incubated at 37 °C for 24 h [30].

### 2.2. EPS Extraction

EPS produced by *S. thermophilus* SBC8781 was extracted according to the previously described ethanol precipitation method [10,11]. For the extraction of EPS from fermented milks, the cultures were centrifuged (8000× *g*, 20 min, 4 °C) to remove bacterial cells and to collect the supernatant. An equal volume of ethanol was added to the supernatant and kept at 4 °C overnight. The precipitate containing EPS was collected by centrifugation (12,000× *g* 10 min), dissolved in distilled water, and treated with DNase (7 μg/mL) and Rnase (7 μg/mL) (Sigma-Aldrich, St. Louis, MO, USA) for 6 h at 37 °C. Later, the samples were treated with proteinase K (200 μg/mL, Sigma-Aldrich, St. Louis, MO, USA) at 37 °C overnight. EPS samples were heated at 100 °C for 10 min to inactivate the enzymes. After cooling, the samples were precipitated by ethanol, dialyzed against distilled water using the membrane MW: 8000–10,000 (Spectrum Laboratories, Rancho Dominguez, CA, USA) for 48 h, and lyophilized.

### 2.3. Anion Exchange Chromatography

The crude EPSs were fractionated by anion exchange column HiTrap^TM^ Q HP (Cytiva, Tokyo, Japan) [10,11]. EPS was applied to the column HiTrap^TM^ Q HP, and the column was eluted with water and linear gradient elution from 0 to 1.0 M of NaCl in 50 mM Tris-HCl buffer (pH 8.6). Two mL of each eluate was monitored by Phenol-H_2_SO_4_ reaction using a total carbohydrate assay kit, according to the manufacturer protocol (Cell BioLabs. Inc, SanDiego, CA, USA).

### 2.4. Gel Chromatography

The crude EPSs were purified by gel chromatography with Superdex^TM^ 200 Increase 10/300GL (Cytiva, Tokyo, Japan) using the isocratic gradient of 0.2 M NaCl/50 mM Tris-HCl (pH 8.6). The total amount of carbohydrates was measured using a total carbohydrate assay kit, as described by the providers (Cell BioLabs. Inc., SanDiego, CA, USA).

### 2.5. Sugar Composition

EPSs produced by *S. thermophilus* SBC8781 were dissolved in mQ water to obtain a solution of 200 µg/mL, and ABEE labeling was performed using an ABEE Labeling kit (J-chemicals, Tokyo, Japan). Then, 50 µL of EPS sample was mixed with 50 µL of 8M TFA, and the mixture was heated at 100 °C for 3 h. After drying with a centrifugal evaporator, 40 µL of pyridine/MeOH (10:90) and 10 µL of acetic anhydride were added, allowed to stand at room temperature for 30 min, and dried again. The dried material was redissolved in 10 µL of mQ water, 40 µL of ABEE labeling reagent was added, and then it was heated at 80 °C for 60 min. Finally, 200 μL of mQ water and 200 μL of chloroform were added, and the mixture was centrifuged at 12,000× *g* for 5 min; the supernatant was collected and used for sugar determinations. The concentration of each sugar constituent was measured with the Agilent 1100 HPLC system (fluorescence detector G1321A FLD) with a column Honepak C18 (75 mm × 4.6 mm) and the eluents 0.2 M potassium borate buffer (pH 8.9)/acetonitrile (93:7) and 0.02% TFA/acetonitrile (50:50).

### 2.6. NMR Spectroscopy

The proton NMR spectra (500.16 MHz, JNM-ECZ500R; JEOL Ltd., Tokyo, Japan) were obtained by the single pulse method with DANTE presatulation. Measurement was performed at a temperature of 30 °C, a pulse of 45°, a pulse repetition time of 7 s, 512 scans, and a measurement time of 1 h. EPS samples were dissolved in D_2_O to a concentration of 2 mg/mL, and TSP-d4 was used as an internal standard.

### 2.7. PIE Cells and Immunomodulatory Assays

The immunomodulatory activity analysis of EPSs was performed according to the previous publications [30,31,32]. The porcine intestinal epithelial (PIE) cell line was derived from intestinal epithelia isolated from newborn unsuckled pig [32]. PIE cells were maintained in high-glucose Dulbecco’s Modified Eagle’s medium (DMEM) (Gibco, Rockville, MD, USA) supplemented with 10% (*v*/*v*) fetal bovine serum (Sigma-Aldrich, St. Louis, MO, USA) and 100 IU/mL streptomycin (Nakalai Tesque, Kyoto, Japan) at 37 °C in a humidified atmosphere of 5% CO_2_. PIE cell cultures were grown in a 250 mL flask and passaged routinely to reach approximately 80–90% confluence.

PIE cells were seeded at 3 × 10^4^ cells/well to a 24-well plate and incubated for 3 days at 37 °C in 5% CO_2_. After 3 days of incubation, cells were washed twice with PBS and stimulated with purified EPS (100 μg/mL) from soy milk (EPS-s) or cow milk (EPS-m) for 48 h. After the treatment, cells were challenged with poly(I:C) (3 μg/mL) (R&D Systems, Minneapolis, MN, USA) for 2 h for RT-PCR studies.

### 2.8. RT-PCR Analysis

Total RNA was extracted and purified by Trizol reagent (Molecular Research Center, Cincinnati, OH, USA) from each PIE sample, and the quality and quantity of total RNA were verified by a Nanodrop spectrometer (Nanodroptechnologies, Wilmington, DE, USA). For the preparation of cDNA, 1 μg of total RNA samples was reverse transcribed by the PrimeScript™ RT reagent Kit (Takara, Shiga, Japan) according to the manufacturer’s instructions. Gene expression was measured by real-time RT-PCR in a Light Cycler 480 (Roche Diagnostics, Mannheim, Germany) using specific primers (Supplementary Table S1) and Platinum SYBR green (Invitrogen, Carlsbad, CA, USA). The total reaction mixture volume was 10 μL, containing 2.5 μL of cDNA and 7.5 μL of master mix, including forward and reverse primers. Amplification was carried out at the following conditions: 50 °C for 2 min and 95 °C for 5 min, followed by 40 cycles of 95 °C for 15 s, 60 °C for 30 s, and 72 °C for 30 s. β-actin was used as an internal standard to normalize cDNA levels for differences in total cDNA levels in the samples.

### 2.9. Comparative mRNA Analysis of Sugar Metabolism and EPS Biosynthesis Genes

*S. thermophilus* SBC8781 was cultured in whey-based medium for EPS production under aerobic conditions at 37 °C. After cultivation, cells were collected by centrifugation and washed twice with PBS. The cDNA was prepared by the PrimeScript™ RT reagent kit. Gene expression was measured by real-time RT-PCR using specific primers (Supplementary Tables S2 and S3), and differentially expressed Morpheus heat maps performed gene expression analysis.

### 2.10. Statistical Analysis

The experimental data were expressed as means and their standard error (SE) and all statistical analyses were performed using the JMP 16.0 program (JMP, Tokyo, Japan). Differences between averages were tested using the Tukey-Kramer method on a significance level of *p* < 0.05. One-way ANOVA was performed, followed by the calculation of the Dunnett’s post hoc test, using the non-stimulated group (negative control) as reference, and a significant difference was accepted as *p* < 0.05.

## 3. Results

### 3.1. Production, Purification, and Partial Characterization of EPSs from Soy Milk and Milk

*S. thermophilus* SBC8781 could grow up and ferment two culture broths of different food origins: vegetable (soy milk) and animal (cow milk). As a result, the SBC8781 strain was able to produce EPS in both culture media. The purified biopolymers from soy milk (EPS-s) and cow milk (EPS-m) showed similar aspects, light white powder. Both biopolymers’ UV spectra showed one band between 190 and 210 nm, typical for carbohydrates. The lack of signals at 260, 280, and 400 nm indicated the sample’s absence of proteins, nucleic acids, and pigments, respectively [33]. The polymer yield in soy milk (200–400 mg/L) was higher than in cow milk (50–70 mg/L). The identity of the isolates as the EPS structure was demonstrated by ^1^H-NMR (Supplementary Figure S1). The chemical shifts from δ 3.0 to 5.5 ppm in the 1H NMR spectrum were assigned to protons of carbon rings and hydroxyl groups in EPS structure. The multiple peaks from 4.8 to 5.5 ppm corresponded to the protons of monomer units in the EPS-s and EPS-m backbones [33].

**Figure 1:**
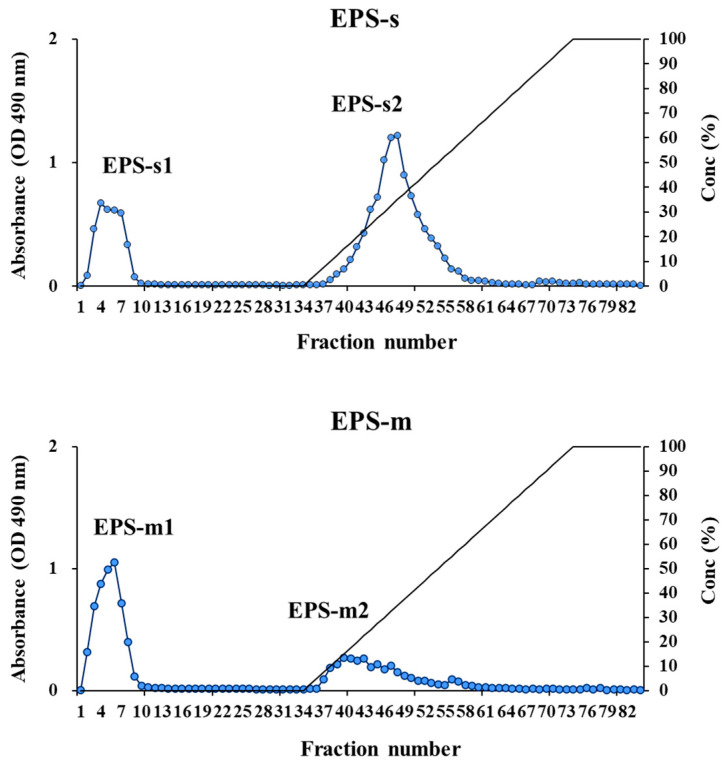
Anion exchange chromatogram of exopolysaccharides isolated from soy milk (EPS-s) and cow milk (EPS-m) after fermentation with *Streptococcus thermophilus* SBC8781.

Anion exchange chromatography was used with purified EPSs to board knowledge of their structure based on charges (neutral or acidic polysaccharides). In Figure 1, the solutions of purified EPSs were applied to a HiTrap^®^ column, resulting in two fractions for each sample (EPS-s and EPS-m) eluted with MiliQ^®^ water and NaCl solutions (0.05–0.70 M). Each fraction was named EPS-s1 and EPS-s2 (ratio: 27.78% and 72.22%) for EPSs and EPS-m1 and EPS-m2 (ratio: 55.43% and 44.57%) for EPS-m. Since the chromatographic column is an anion exchanger with positive charges, it could bind to anionic molecules in the buffer in the balancing process. Therefore, neutral groups were removed with water as eluent, and negatively charged groups were extracted with NaCl solutions. For the above, EPS-s1 and EPS-m1 were neutral polysaccharides, while EPS-s2 and EPS-m2 were ascribed to the acidic polymer type. Additionally, EPS-s possessed higher quantities of acidic polysaccharides than EPS-m.

**Figure 2:**
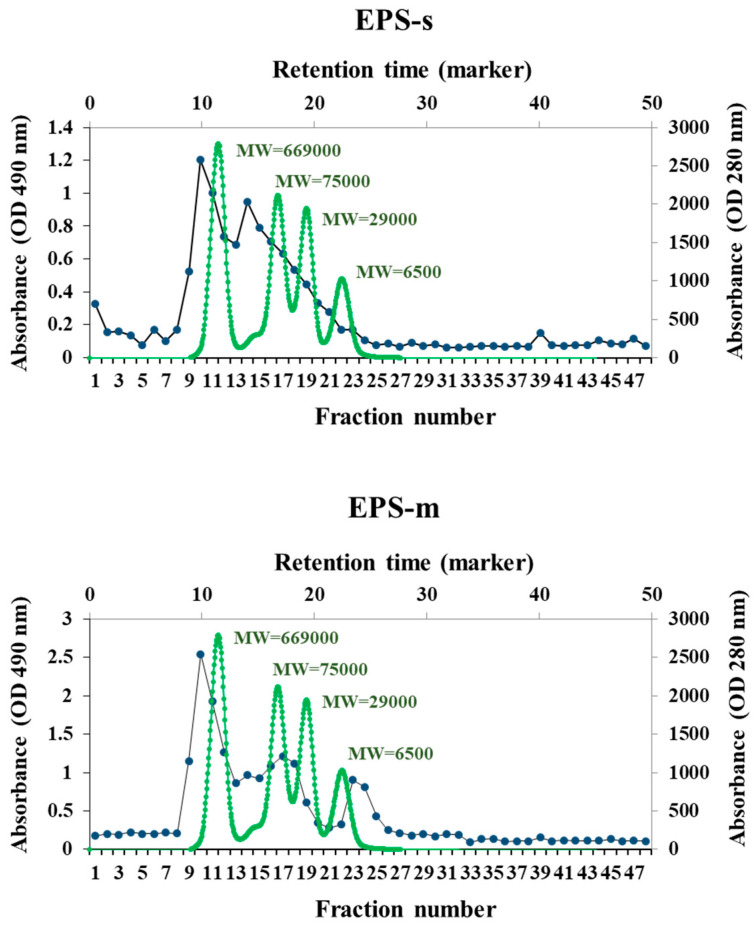
Gel filtration chromatogram of exopolysaccharides isolated from soy milk (EPS-s) and cow milk (EPS-m) after fermentation with *Streptococcus thermophilus* SBC8781.

The average molecular weight (Mw) of the purified EPSs was determined by gel permeation chromatography. Figure 2 shows non-symmetrical profiles of the carbohydrate detection of each chromatography fraction obtained by EPS-s and EPS-m. In this context, EPS-m exhibited three main signals between 2.4 and 1890 kDa, with an average Mw of 1108, 139, and 4.2 kDa, respectively. By contrast, EPS-s had two significant peaks between 5.6 and 1890 kDa, with an average Mw of 1074 kDa and 133 kDa, respectively. These results suggest that the SBC8781 strain produced polysaccharides of similar average Mw in soy milk and cow milk. However, the EPS made from vegetable-origin broth exhibited apparent populations of polymers of higher Mw than EPS produced from the animal-origin broth.

**Figure 3:**
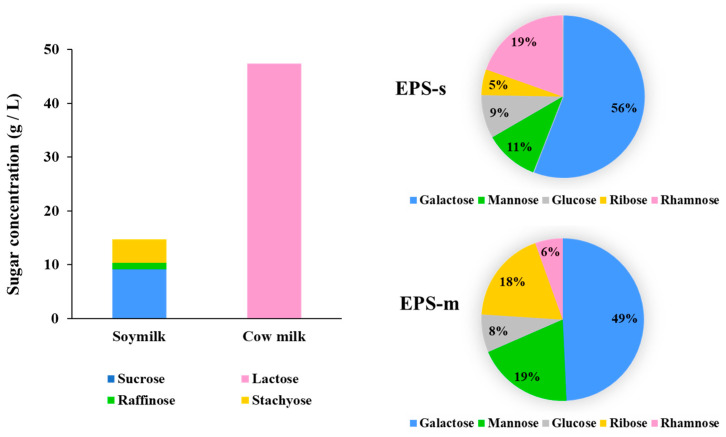
Sugar concentration of soy milk and cow milk (bar graphs) and sugar composition of exopolysaccharides isolated from soy milk (EPS-s) and cow milk (EPS-m) after fermentation with *Streptococcus thermophilus* SBC8781 (pie charts).

The monomer composition of purified EPSs and the sugar composition of both culture broths were analyzed. The results are presented in Figure 3. The monomer composition studies of both EPS produced by the SBC8781 strain demonstrated that they belonged to the heteropolysaccharide-type (HePS, EPS composed of two or more monosaccharides) [34]. Galactose, rhamnose, mannose, glucose, and ribose were detected in both purified polymer samples, although with different ratios, 0.56:0.19:0.11:0.09:0.05 and 0.49:0.06:0.19:0.08:0.18 for EPS-s and EPS-m, respectively. Galactose was the most abundant monomer, while glucose was proportionally similar in both polymer structures. However, rhamnose, mannose, and ribose contents exhibited opposite trends in the chemical EPS-s and EPS-m structures. In this sense, the carbon and nitrogen sources between both culture media could explain why *S. thermophilus* SBC8781 produced polysaccharides with different chemical structures and yields. Sucrose and stachyose were in large quantities in soy milk (9.5 and 4.5 g/L, respectively), while lactose was the primary sugar in cow milk (47.5 g/L). Then, the sugar differences as carbon and energy sources in both culture broths of different origins could explain why *S. thermophilus* SBC8781 produces EPS with varying structures of chemicals and yields.

### 3.2. Transcriptomic Analysis of Sugar Metabolism and EPS Biosynthesis Genes in S. thermophilus SBC8781

**Figure 4:**
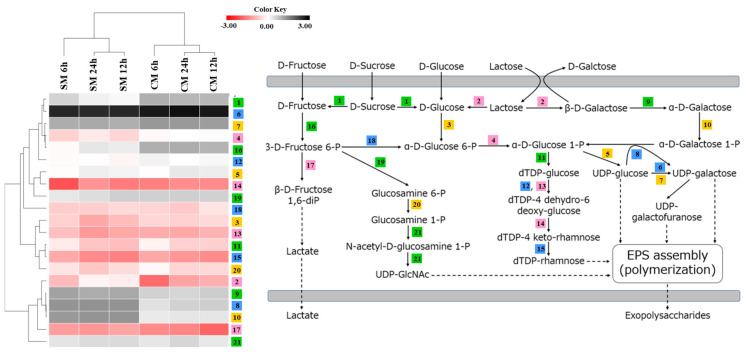
Comparative gene expression level of nucleotide sugar synthesis-associated genes in *Streptococcus thermophilus* SBC8781 growing in soy milk (SM) or cow milk (CM) for 6, 12, and 24 h. Heatmap expression level of nucleotide sugar synthesis associated genes and nucleotide synthesis-related biosynthesis pathways. 1: sucrose-6-phosphate hydrolase, 2: β-galactosidase, 3: glucokinase, 4: phosphoglucomutase, 5: UTP-glucose-1-phosphate uridyltransferase GalU, 6: UDP-glucose 4 epimerase, 7: UDP-galactose 4 epimerase, 8: galactose-1-phosphate uridylyltransferase, 9: galactose mutarotase, 10: galactokinase, 11: dTDP-glucose pyrophosphorylase, 12: dTDP glucose-4, 6-dehydratase, 13: dTDP-4-dehydrorhamnose 3.5-epimerase, 14: dTDP-4 dehydrorhamnose 3.5-epimerase, 15: dTDP-4 keto-L-rhamnose reductase, 16: fructokinase, 17: 6-phosphofructokinase, 18: phosphoglucose isomerase, 19: glutamine-fructose-6-phosphhate transaminase, 20: phosphoglucosamine mutase, 21: N-acetylglucosamine-1-phosphate uridyltransferase, and 22: UDP-galactopyranose mutase.

Since the EPS-s and EPS-m produced by *S. thermophilus* SBC8781 may differ in structure and composition, we investigated the expression levels of genes related to sugar metabolism and EPS synthesis in the SBC8781 strain when growing in soy milk or cow milk. First, genes related to sugar metabolism were examined for changes in expression over a time of 6, 12, and 24 h (Figure 4). In soy milk, sucrose-6-phosphate hydrolase (*scrB*), phosphoglucomutase (*pgmA*), UTP-glucose-1-phosphate uridyltransferase (*cap4C*), and fructokinase (*scrK*) expression levels were increased compared to those in cow milk. In contrast, the expression levels of β-galactosidase (*lacZ*), galactose-1-phosphate uridyltransferase (*galT*), galactose mutarotase (*galK*), and galactokinase (*galR*) levels were increased in cow milk compared to those in soy milk (Figure 4).

**Figure 5:**
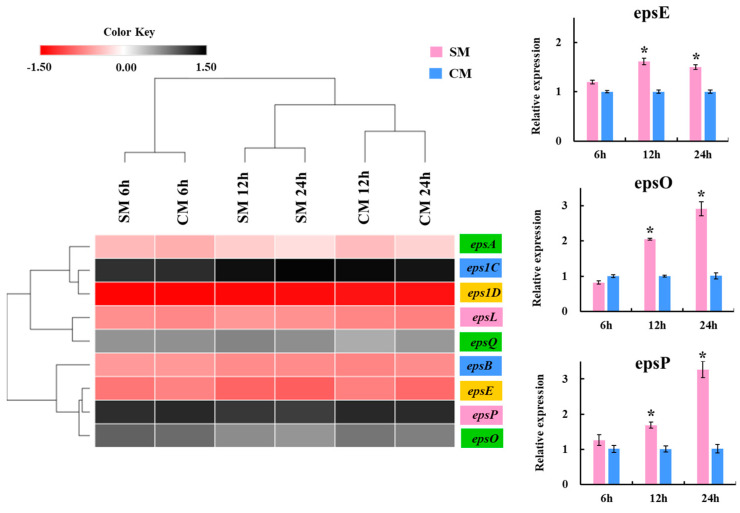
Heatmap of expression levels of exopolysaccharide assembly-associated genes in *Streptococcus thermophilus* SBC8781 growing in soy milk (SM) or cow milk (CM) for 6, 12, and 24 h. Expression of *epsE*, *epsO*, and *epsP* genes. Asterisks indicate significant differences when compared to the CM, * *p* < 0.05.

In addition, changes in the expression levels for the EPS synthesis gene cluster were evaluated over time periods of 6, 12, and 24 h. It was described that a total of 20 genes related to EPS biosynthesis are expressed in the species *S. thermophilus* [35]. Here, only nine genes related to EPS biosynthesis (*epsA*, *epsB*, *eps1C*, *eps1D*, *epsE*, *epsL*, *epsO*, *epsP*, and *epsQ*) were identified in the SBC8781 strain (Figure 5). A comparison of expression levels was performed for these nine EPS synthesis genes in soy milk and cow milk. A tendency for higher expression levels was detected for *epsA*, *epsB*, *eps1C*, and *eps1D* in soy milk compared to cow milk. In addition, significantly higher expressions were observed for *epsE*, *epsO*, and *epsP* in soy milk than in cow milk at hours 12 and 24 (Figure 5).

### 3.3. Immunomodulatory Activities of EPSs Produced by S. thermophilus SBC8781 in Soy Milk and Cow Milk

**Figure 6:**
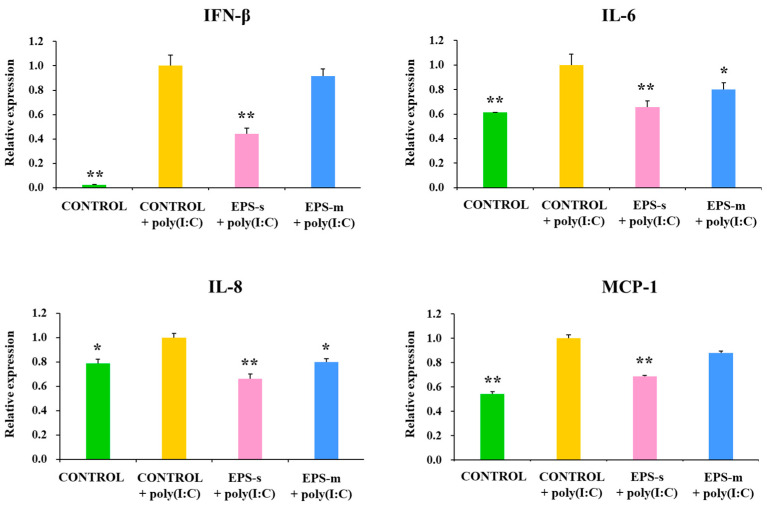
The effect of exopolysaccharides isolated from soy milk (EPS-s) and cow milk (EPS-m) after fermentation with *Streptococcus thermophilus* SBC8781 based on the relative expression levels of *IFN-β*, *IL-6*, *IL-8*, and *MCP-1* genes in porcine intestinal epithelial (PIE) cells stimulated with poly(I:C). Unstimulated PIE cells and cells treated only with poly were used as controls. Asterisks indicate significant differences when compared to the poly(I:C) stimulated control group, * *p* < 0.05; ** *p* < 0.01.

**Figure 7:**
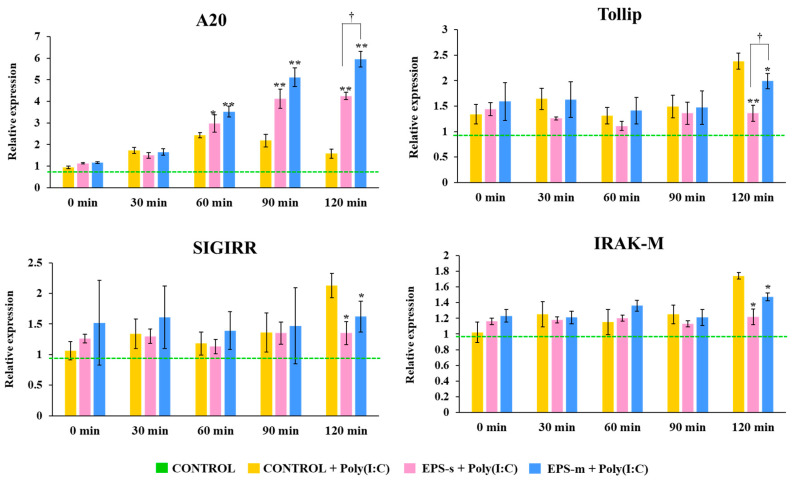
The effect of exopolysaccharides isolated from soy milk (EPS-s) and cow milk (EPS-m) after fermentation with *Streptococcus thermophilus* SBC8781 based on the relative expression levels of *A20*, *Tollip*, *SIGIRR*, and *IRAKM* genes in porcine intestinal epithelial (PIE) cells stimulated with poly(I:C). Unstimulated PIE cells and cells treated only with poly were used as controls. Asterisks indicate significant differences when compared to the poly(I:C) stimulated control group, * *p* < 0.05; ** *p* < 0.01. ^†^ Significant differences between the indicated groups, * *p* < 0.05.

In the next set of experiments, we aimed to evaluate whether EPS-s and EPS-m were able to modulate the innate antiviral immune response in PIE cells. For this purpose, EPS-s and EPS-m were used in a similar concentration (100 µg/mL) for the stimulation of PIE cells followed by the challenge with the TLR3 agonist poly(I:C). As shown in Figure 6, the activation of the TLR3 signaling pathway in PIE cells significantly increased the expression of the inflammatory cytokines *IFN-β* and *IL-6* as well as the chemokines *IL-8* and *MCP-1*. EPS-s was able to reduce the expression of all the inflammatory factors evaluated in PIE cells after the stimulation of poly(I:C). Similarly, cells treated with EPS-m showed significantly lower levels of *IL-6* and *IL-8* compared to the poly(I:C)-challenged control cells, while no modification was observed for *IFN-β* and *IL-6* (Figure 7). Interestingly, the effect of EPS-s in the expression of *IL-6* and *IL-8* was more remarkable than EPS-m.

We also evaluated the effect of EPSs produced by *S. thermophilus* SBC8781 on the expression of the following negative regulators involved in the control of the TLR3 signaling pathway activation: *A20*, *Bcl-3*, *SIGIRR*, *Tollip*, *MKP-1*, and *IRAK-M* (Figure 7, Supplementary Figure S2). PIE cells were treated with EPS-s or EPS-m and then stimulated for 120 min with poly(I:C). The challenge of PIE cells with poly(I:C) significantly increased the expression of *A20* and *MKP-1* from minute 30 after stimulation, while the expressions of *Bcl-3*, *SIGIRR*, *Tollip*, and *IRAK-M* were augmented from minute 120. No significant changes were found when the expressions of *Bcl3* and *MKP-1* in EPS-s- and EPS-m-treated cells were compared to controls (Supplementary Figure S1). Both EPS-s and EPS-m significantly enhanced the expression of *A20* in PIE cells from minute 60. The expression levels of this negative regulator only differed from each other at minute 120 (Figure 7). In contrast, the expression levels of *Tollip*, *SIGIRR*, and *IRAK-M* in PIE cells treated with EPS-s and EPS-m were lower than controls at minute 120. Differences between EPS-s and EPS-m were only observed in the expression of *Tollip* (Figure 7).

## 4. Discussions

*S. thermophilus* is a homofermentative thermophilic LAB, widely used as a starter culture in homemade and industrial-fermented dairy products. Some *S. thermophilus* strains can synthesize EPS, which received particular attention over recent decades since they improve the texture and mouthfeel of fermented food matrices and confer beneficial health properties, including gastroprotection, antioxidant activities, and immunomodulatory functions [18,36,37,38,39]. The current report aimed to evaluate EPS production as well as to isolate and partially characterize the biopolymers produced by *S. thermophilus* SBC8781 in two food-origin culture broths. Furthermore, the EPS biosynthesis gene expression of the SBC8781 strain and the biopolymers’ capacity to modulate antiviral immunity in intestinal epithelial cells were evaluated.

Our results suggest that *S. thermophilus* SBC8781 produced biopolymers identified as EPS by 1H-NMR in soy milk (EPS-s, 200–400 mg/L) and cow milk (EPS-m, 50–70 mg/L) with different yields. The isolated EPSs had high purity levels, and they exhibited proportional differences in monomers, charged structures, and Mw. In this sense, EPS-s showed more polymer populations with high Mw and acidic structures than EPS-m. This notable variation was probably due to the differences in nutritional compositions (protein, carbohydrates, vitamins, etc.) of vegetable- and animal-based media. In this context, a similar behavior was reported for *S. thermophilus* 05–34, which produced a higher EPS concentration in milk-based broth supplemented with soy protein (~150 mg/L) than whey protein (~100 mg/L) [36]. Moreover, the work described that the 05–34 strain incremented the average Mw of the EPS produced in milk supplemented with soy protein (470 kDa) compared to whey protein (25 kDa). The monomer analysis of EPS-s and EPS-m showed that both belonged to the HePS classification composed of galactose, rhamnose, mannose, glucose, and ribose, although with different ratios. Galactose was the most abundant monomer, while glucose was proportionally similar in both polymer structures. Vaningelgem et al. [40] reported similar results for several HePS results produced by *S. thermophilus* strains. Of note, sucrose and stachyose were the highest carbon sources in soy milk (9.5 and 4.5 g/L, respectively), while lactose was the primary sugar in cow milk (47.5 g/L). The EPS biosynthetic pathways in *S. thermophilus* consist of sugar transport from the environment into the cytoplasm, sugar-1-phosphate synthesis, polysaccharide synthesis, and export to the extracellular sites as macromolecules via a particular lipid carrier [34]. Then, it is possible to speculate that the differences between soy milk and cow milk in sugars as carbon and energy sources could explain why *S. thermophilus* SBC8781 produces EPS with varying structures of chemicals and yields. In fact, higher expressions of *lacZ*, *galT*, *galK*, and *galR*, which are genes involved in the metabolism of lactose, were observed in the SBC8781 strain growing cow milk compared to soy milk. In contrast, *scrB* and *scrK*, which are enzymes involved in sucrose metabolism, were significantly upregulated in soy milk. We also detected differences in the expressions of genes related to EPS production. The bacterial biosynthesis of EPS is a complex biochemical process regulated by the *eps* gene cluster located on the chromosome or plasmid [5]. A study by Makino et al. [35] reported that 20 *eps* genes in the genome of *S. thermophilus* were involved in the biosynthesis of EPS. In this study, 9 *eps* genes were identified in the genome *S. thermophilus* SBC8781, including genes involved in the regulation of EPS production (*epsA* and *epsB*), chain length determination (*epsC*, *epsD*), the biosynthesis of repeating sugar units (*epsE*), polymerization, and the export of repeating units (*epsO*, *epsP*, *epsQ*) [35,37,38]. A tendency for higher expression levels of *epsA*, *epsB*, *eps1C*, and *eps1D* genes and significantly higher expression levels of *epsE*, *epsO*, and *epsP* genes were found in soy milk compared to cow milk, which is in line with the higher EPS production observed for the SBC8781 strain in soy milk.

In addition to quantitative differences, qualitative differences could be observed when comparing EPSs produced by *S. thermophilus* SBC8781. Both EPS-s and EPS-m are acidic polysaccharides and have similar Mw. However, a higher number of acidic groups were found in EPS-s than EPS-m. Probably, the oxidized rates of transforming primary hydroxyl groups of sugars to form acidic groups were high during fermentation, which accounted for more acidic polysaccharides in EPS-s. It was reported that the oxidation of hydroxyl groups in glucose are responsible for forming acidic groups in polysaccharides during the growth of *S. thermophilus* CH9 in skim milk-based medium [39]. Furthermore, the structural composition analysis of EPSs produced by *S. thermophilus* SBC8781 demonstrated that both EPS-s and EPS-m were heteropolysaccharides containing different monosaccharides arranged in groups. Galactose was the principal component present in EPS-s and EPS-m. In line with these results, it was reported that galactose was the most abundant sugar in EPSs produced by 26 strains of *S. thermophilus* after the fermentation of skim milk [40]. Similarly, galactose and glucose were described as the most common monosaccharides in EPS derived from *S. thermophilus* [14,41]. In addition to galactose and glucose, mannose, ribose, and rhamnose were also found in both EPS-s and EPS-m. The presence of these monosaccharides in EPS produced by *S. thermophilus* ST1 during skim milk fermentation were described by Zhang et al. [42]. Although several analytical methods were used to study the EPS molecules produced by the SBC8781 strain, there is not enough information to propose definitive structures for EPS-s and EPS-m. Further studies are needed to determine the differences in the structures of both EPSs.

In general, EPS structures and properties vary depending on media ingredients and culture conditions [43]. The EPS structural characteristics of charge, linearity, and glycosidic bond can notably affect the texture, stability, water holding capacity, and sensory properties of fermented milk [5]. Of note, the structural differences in EPS molecules were reported to have an impact not only regarding their rheological and physical properties [5] but also regarding their functional activities [4,44,45]. Thus, in this study, we also aimed to examine whether EPS-s and EPS-m derived from *S. thermophilus* SBC8781 have different immunomodulatory activities in the context of TLR3-triggered intestinal innate antiviral immunity. Upon meeting with viral pathogens, intestinal epithelial cells initiated mucosal immune responses to prevent infection by activating innate pathways, such as the TLR3 signaling pathway, which stimulate the production of type I IFNs and inflammatory cytokines that recruit and activate immune cells [32]. In line with our previous results demonstrating that poly(I:C) activates TLR3-IRF3 signaling in intestinal epithelial cells leading to the upregulation of proinflammatory factors [11,31], we observed here that the expressions of *IFN-β*, *IL-6*, *IL-8*, and *MCP-1* were upregulated in PIE cells. We also observed that both EPSs produced by *S. thermophilus* SBC8781 were able to reduce the expression of *IL-6* and *IL-8* in PIE cells stimulated with poly(I:C), being the effect of more-pronounced EPS-s than that observed for EPS-m. Moreover, only EPS-s was able to reduce the expression of *IFN-β* and *MCP-1* in poly(I:C)-challenged PIE cells. These results are in line with our previous findings demonstrating that the EPSs from *L. delrueckii* OLL1073R-1 [32] were able to differentially regulate the expression of inflammatory factors in PIE cells after stimulation with poly(I:C). Interestingly, the immunoregulatory effects of EPSs derived from the OLL1073R-1 strain were related to their ability to modulate the expression of several negative regulators of the TLR signaling pathway. Thus, we also evaluated here whether EPS-s and EPS-m could induce similar changes. In our hands, both EPSs produced by *S. thermophilus* SBC8781 differentially regulated the expression levels of *A20*, *Tollip*, *SIGIRR*, and *IRAK-M*. Of note, the upregulation of *A20* was more remarkable for EPS-m treatment, while the down-regulation of *Tollip* was more noticeable for EPS-s. The different relative changes induced in the set of negative regulators could explain the different effects found in the expression of inflammatory mediators when EPS-s and EPS-m were compared. More in-depth molecular studies on the TLR3-IRF3 signaling pathway are necessary to conclusively explain the different effect of both EPSs. The elucidation of the precise structures of EPS-s and EPS-m could also contribute to understanding their immunomodulatory differences, since it would make it possible to predict their interactions with immunological receptors involved in intestinal innate immunity. In this regard, we demonstrated previously that the acidic EPS produced by *Lactiplantibacillus plantarum* N14 modulated the inflammatory response of PIE cells stimulated with LPS, while this property was not observed for its neutral EPS [4]. Acidic EPS from the N14 strain exerted its immunomodulatory activities in PIE cells in a RP105/MD1-dependent manner [4]. Then, considering that the number of acidic polysaccharides was higher in EPS-s compared to EPS-m, it could be speculated that EPS-s has a greater capacity to modulate signaling pathways, such as the RP105/MD1, and thus exert a more prominent immunomodulatory effect. In addition, in vivo studies comparing the immunomodulatory effects of EPS-s and EPS-m could be of value to effectively demonstrate the advantages of EPS produced by *S. thermophilus* SBC8781 in soy milk. These are studies that we intend to perform in the immediate future.

## 5. Conclusions

EPSs produced by *S. thermophilus* SBC8781 were isolated and purified from fermented soy milk and cow milk. Moreover, their abilities to modulate TLR3-mediated immunity in intestinal epithelial cells were characterized. The results showed that the SBC8781 strain produced different amounts of EPS in vegetable- and animal-based media, although both showed a high purity grade. EPS-s and EPS-m were identified as polysaccharides of the HePS-type, formed by galactose, glucose, rhamnose, ribose, and mannose and with similar structural aspects. However, EPS-s showed notable differences with monomer proportion and more polymer populations with high Mw and acidic structures than EPS-m. In vitro studies in PIE cells demonstrated the immunomodulatory properties of both EPS-s and EPS-m in the context of TLR3 activation. Of note, the effect of EPS-s was more remarkable than that observed for EPS-m. The higher production of EPS in soy milk and its better immunomodulatory activity makes EPS-s from *S. thermophilus* SBC8781 an attractive functional food component that should be further evaluated in terms of its technological and immunomodulatory properties. Furthermore, *S. thermophilus* SBC8781 could be used as an immunobiotic strain to develop new immunologically functional soy milk-based foods with health-beneficial properties, which might help improve protection against viral infections. The isolation and the structural and macromolecular characterization of the neutral and acidic polymer fractions of EPS-s and EPS-m are essential to expand our understanding of the relationship between structure and immune function. Research on the isolation and structural and macromolecular characterization of each biopolymer’s neutral and acidic fractions and their immunomodulatory effects is underway for a more profound knowledge of the relationship between structure and immune function.

## Data Availability

Data is contained within the article.

## Supplementary Materials

The following supporting information can be downloaded at: https://www.mdpi.com/article/10.3390/foods12122374/s1, Figure S1: 1H-NMR spectra of exopolysaccharides isolated from soy milk (EPS-s) and cow milk (EPS-m) after fermentation with *Streptococcus thermophilus* SBC8781. Figure S2: The effect of exopolysaccharides isolated from soy milk (EPS-s) and cow milk (EPS-m) after fermentation with *Streptococcus thermophilus* SBC8781 on the relative expression levels of *Bcl3* and *MKP-1* genes in porcine intestinal epithelial (PIE) cells stimulated with poly(I:C). Unstimulated PIE cells and cells treated only with poly were used as controls. Table S1: List of primers used in this study for cytokines, chemokines, and negative regulators. Table S2: List of primers used in this study for sugar metabolism genes. Table S3: List of primers used in this study for exopolysaccharides biosynthesis genes.
