# Supplementary material for: Partial Characterization and Immunomodulatory Effects of Exopolysaccharides from Streptococcus thermophilus SBC8781 during Soy Milk and Cow Milk Fermentation
Source: Foods. 2023 Jun 15;12(12):2374. doi: 10.3390/foods12122374 (PMC10297478; doi:10.3390/foods12122374)
Supplement: Supplementary file 1 [file foods-12-02374-s001.zip › foods-2447069-supplementary.pdf]

Figure S1. ^{1}H-NMR spectra of exopolysaccharides isolated from soymilk (EPS-s) and cow milk (EPS-m) after the fermentation with *Streptococcus thermophilus* SBC8781.

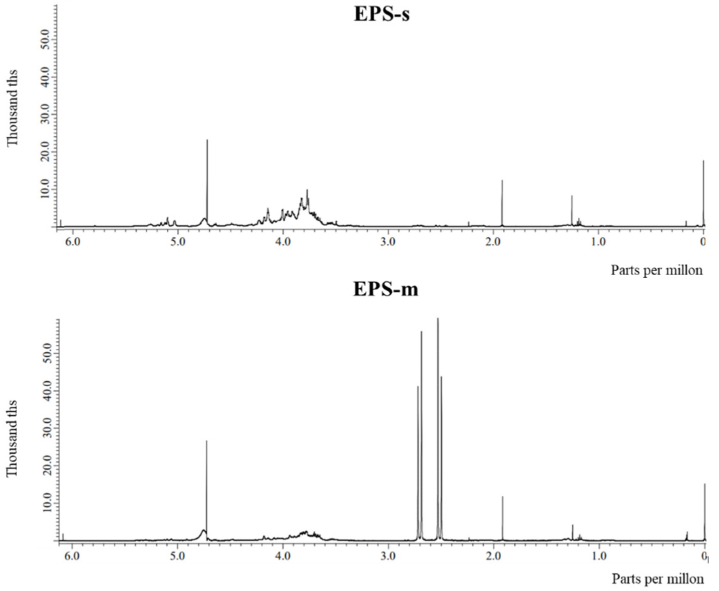

Figure S2. Effect of exopolysaccharides isolated from soymilk (EPS-s) and cow milk (EPS-m) after the fermentation with Streptococcus thermophilus SBC8781 on the relative expression levels of *Bcl3* and *MKP-1* genes in porcine intestinal epithelial (PIE) cells stimulated with poly(I:C). Unstimulated PIE cells and cells treated only with poly were used as controls.

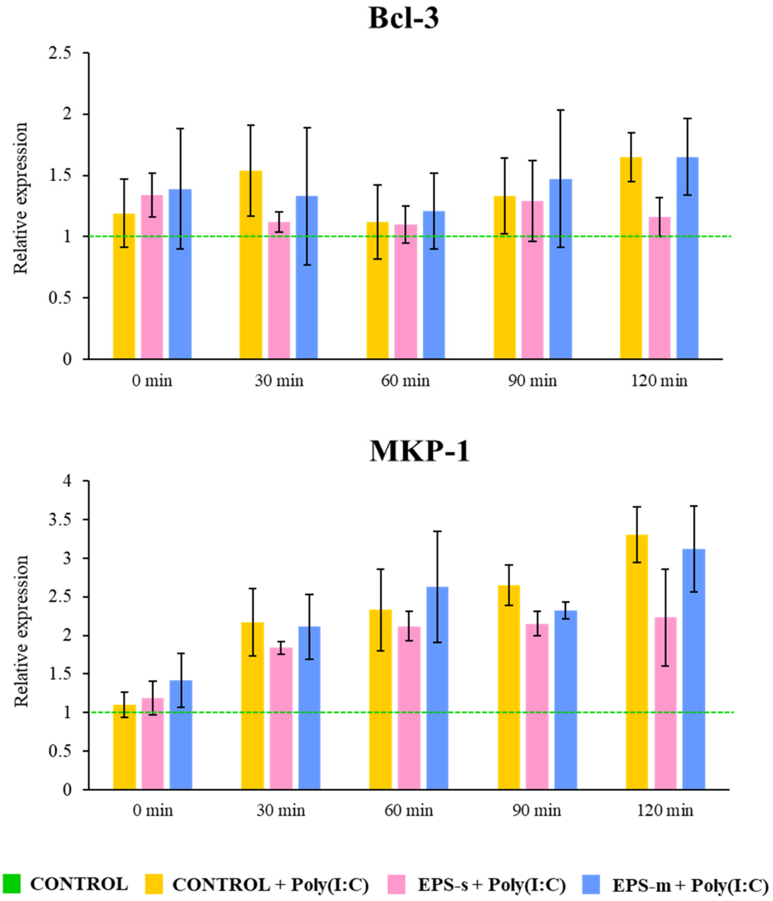

Table S1. List of primers used in this study for cytokines, chemokines and negative regulators

Gene	Primer	Sequence (5'→3')
β-Actin	Forward	CATCACCATCGGCAACGA
	Reverse	GCGTAGAGGTCCTTCCTGATGT
IL-6	Forward	TGGATAAGCTGCAGTCACAG
	Reverse	ATTATCCGAATGGCCCTCAG
IL-8	Forward	GCTCTCTGTGAGGCTGCAGTT
	Reverse	TTTATGCACTGGCATCGAAGTT
MCP-1	Forward	ACAGAAGAGTCACCAGCAGCAA
	Reverse	GCCCGCGATGGTCTTG
IFN-β	Forward	AGTTGCCTGGGACTCCTCAA
	Reverse	CCTCAGGGACCTCAAAGTTCAT
A20	Forward	CCTCCCTGGAAAGCCAGAA
	Reverse	GTGCCACAAGCTTCCTCACTT
Bcl-3	Forward	CGACGCGGTGGACATTAAG
	Reverse	ACCATGCTAAGGCTGTTGTTTTC
SIGIRR	Forward	ATGTGAAGTGTCGGCTCAATGT
	Reverse	TTCATCTCCACCTCCCCATA
Tollip	Forward	TACCGTGGGCCGTCTCA
	Reverse	CCGTAGTTCTTCGCCAACTTG
MKP-1	Forward	AACGAGGGTCAGGCTTTTCC
	Reverse	TCCCCAATGTGCTGAGTTCAG
IRAKM	Forward	TGGAGCAGCCTTGAATCCTT
	Reverse	TGGATAACACGTTTGGGAATCTT

Table S2. List of primers used in this study for sugar metabolism genes

Gene	Primer	Sequence (5'→3')
Tuf	Forward	TAACGTCGGTGTCCTTCT
	Reverse	GACGTCCACCTTCTTCTTTAG
Sucrose-6-phosphate hydrolase	Forward	CTGGCGAACAGTACGCTACA
	Reverse	ACCAGAAACGTTCCCTGATG
β-galactosidase	Forward	GCTTGTGTTCTGAGGGAAGC
	Reverse	GCACGTGAAAGCAAGATTGA
Glucokinase	Forward	TTGCGTAGTCGTGTTGAAGG
	Reverse	TGATTCCTGCATCATTTCCA
Phosphoglucomutase	Forward	CAGAAAGCTACGGTGCAACA
	Reverse	CGCGTACAAATGGTTTGATG
UTP-glucose-1-phosphate uridylyltransferas	Forward	GGAGATGCTGTTCTCCAAGC
	Reverse	CATTTTCGCCTTTACCCTGA
UDP-glucose 4 epimerase	Forward	GTCCACCCAGACGCTGTATT
	Reverse	TCCGTAAGTTGCTGCTGTTG
UDP-galactose 4 epimerase	Forward	GTTCACGTCAGACCCCACTT
	Reverse	ATTTAGCTAGGGCTGCACGA
Galactose-1-phosphate uridyl transferase	Forward	AACCAATGCACATCAGTCCA
	Reverse	GGCGACCACCTTGATAGTGT
Galactose mutarotase	Forward	ATTTATGTGCCTGCCAGTCC
	Reverse	ACATCACCAGGAAAGCCATC
Galactokinase	Forward	AACGGATCAGGCTTGTCATC
	Reverse	TGAGGTCAAGGGGTACCAAG
dTDP-glucose pyrophosphorylase	Forward	TGGCTGGAATCAAGGAAATC
	Reverse	AAAACGTGGAAGGTCCTGTG
dTDP-glucose-4, 6-dehydratase	Forward	GAAACGTCCGTGACTGGATT
	Reverse	CACCGATACGCCCCTTAGTA
dTDP-4-dehydrorhamnose 3,5-epimerase	Forward	AAGGATCAGCTTTCGGTGTC
	Reverse	GAGAAAGTGCTTGCCCTGAC
dTDP-4-dehydrorhamnose 3,5-epimerase	Forward	TGCACTTGGTCTTTCAGGTG
	Reverse	ATATACGGCTTGCCTGTTGC
dTDP-4 keto-L-rhamnose reductase	Forward	TTGCGGAAGTAAAACCAACC
	Reverse	GCAGCCTTAGCGACATTTTC
Fructokinase	Forward	TTGGAAGGAATCGCTATTGG
	Reverse	GTGGTTTTGGCGTTGAAGTT
6-phosphofructokinase	Forward	GGCACTTTCCTTCAGTCTGC
	Reverse	GCTGGGAAACCATGTTCAGT
Phosphoglucose isomerase	Forward	TGGACTGGGTTACCTCCAAG
	Reverse	GGCACGTCACCATCTGTATG
Glutamine-fructose-6-phosphhate transaminase	Forward	CAATGCTTCTTCATGCTGGA
	Reverse	CTTCACCGACTGCTTTAGCC
Phosphoglucosamine mutase	Forward	TGGTTCGTCCAAGTGGTACA
	Reverse	TCATCTGTTGGTGCTTCTGC
N-acetylglucosamine-1-phosphate uridyltransferase	Forward	ACTGAACAGTTGGGGACTGG
	Reverse	CGGCAGTTGCAGTCAAGATA

Table S3. List of primers used in this study for exopolysaccharides biosynthesis genes

Gene	Primer	Sequence (5'→3')
epsA	Forward	CCGATTTCAACAGTGTCACG
	Reverse	ATGGACTGTCACACCACCAA
epsB	Forward	CCCAGGGGGTACGTAAGATT
	Reverse	CCAAGATGTGCGAGCACTAA
eps1C	Forward	GATGTCACAACGCTCGAAGA
	Reverse	TTCCAAGCACATCTTCCACA
eps1D	Forward	TCTGGTCCTGTTGCACCTAA
	Reverse	ACCAAAAGACTGGCATCAGC
epsE	Forward	TCGTCGCATTGAAATTGGTA
	Reverse	GGACAATGCTTCCTTGGGTA
epsL	Forward	GTCGGCAATTTCGATTCCTA
	Reverse	ACCTATTGCGTTGCTCCAAG
epsO	Forward	TGGGCTGGAGGATTTAGATG
	Reverse	CTTGTGCATGCCTTTTTGAA
epsQ	Forward	GTGGTTCTACCGGACTTCCA
	Reverse	GCAACTGCTTTGGCTTTTTC

major
